# Increased frequency of self-fertile isolates in *Phytophthora infestans* may attribute to their higher fitness relative to the A1 isolates

**DOI:** 10.1038/srep29428

**Published:** 2016-07-07

**Authors:** Wen Zhu, Lin-Lin Shen, Zhi-Guo Fang, Li-Na Yang, Jia-Feng Zhang, Dan-Li Sun, Jiasui Zhan

**Affiliations:** 1 Fujian Key Lab of Plant Virology, Institute of Plant Virology, Fujian Agriculture and Forestry University, Fuzhou, Fujian, 350002, P. R. China; 2 Xiangyang Academy of Agricultural Sciences, Xiangyang 441057, Hubei, P. R. China; 3 Key Lab for Biopesticide and Chemical Biology, Ministry of Education, Fujian Agriculture and Forestry University, Fuzhou, Fujian, 350002, P. R. China

## Abstract

Knowledge of population dynamics of mating types is important for better understanding pathogen’s evolutionary potential and sustainable management of natural and chemical resources such as host resistances and fungicides. In this study, 2250 *Phytophthora infestans* isolates sampled from 61 fields across China were assayed for spatiotemporal dynamics of mating type frequency. Self-fertile isolates dominated in ~50% of populations and all but one cropping region with an average frequency of 0.64 while no A2 isolates were detected. Analyses of 140 genotypes consisting of 82 self-fertile and 58 A1 isolates indicated that on average self-fertile isolates grew faster, demonstrated higher aggressiveness and were more tolerant to fungicides than A1 isolates; Furthermore, pattern of association between virulence complexity (defined as the number of differential cultivars on which an isolate can induce disease) and frequency was different in the two mating types. In A1 isolates, virulence complexity was negatively correlated (r = −0.515, p = 0.043) with frequency but this correlation was positive (r = 0.532, p = 0.037) in self-fertile isolates. Our results indicate a quick increase of self-fertile isolates possibly attributable to their higher fitness relative to A1 mating type counterpart in the field populations of *P. infestans* in China.

Fungal and fungus-alike pathogens such as *P. infestans* exhibit a wide range of reproductive systems^1^,^2^ including asexual reproduction, parasexual reproduction and sexual reproduction to transmit genetic information across generations. In heterothallic pathogens, sexual reproduction involves nuclear fusion of two distinct strains of opposite mating types, whereas homothallic pathogens are able to undergo this reproductive process within the same strains through selfing, though switches between homothallism and heterothallism have been reported in some pathogens^1^. The reproductive strategies adopted by plant pathogens play important roles in the evolution, epidemics and management of plant diseases through their direct or indirect impact on the generation and maintenance of genetic variation, efficiency of natural selection, extent of genetic drift^3^, competitive ability of immigrants and formation of spores varying in dispersal ability and stress tolerance^4^,^5^,^6^. For example, pathogens with a mixed reproduction system (multiple cycles of asexual multiplication mixed with fewer cycles of sexual reproduction) have a higher evolutionary potential and are more difficult to manage because this reproductive strategy allows the generation of new allelic combinations that may offer an advantage on novel hosts or in changing environments^4^,^7^, while maximizing the preservation of allelic combinations that are well adapted to existing hosts and environments.

The reproductive mode of a particular pathogen is determined not only evolutionarily by mating type genetics (e.g. heterothallism verses homothallism) but also ecologically by the spatiotemporal distribution of mating types in a population. Knowledge of mating type frequency and its spatiotemporal distribution can be used to determine the mating history of pathogen populations and is important for sustainable management of resistance genes and fungicides. In addition to their sexual function, mating type genes may contribute to other biological processes such as aggressiveness, mycelium growth and maintenance of cell wall integrity^8^,^9^,^10^. By combining ecological and life history trait data of pathogens (such as aggressiveness and fungicide tolerance), empirical studies of the frequency distribution of mating types may also inform their quantitative contribution to these biological processes, which usually depend on the genetic background of particular strains and are difficult to be determined by molecular analysis of individual genotypes alone.

Potato (*Solanum tuberosum L*.) is the third-ranked food crop in total production globally^11^. China is the world-leading producer with annual planting areas of >5 million hectares^12^ and the production in the country is expected to expand in the next decades due to changes in social structure, dietary habits and governmental promotion. In China potato production occurs in four major zones: the Northern Single-crop Region (NSR), the Central Double-crop Region (CDR), the Southern Winter-crop Region (SWR) and the Southwestern Multiple-cropping Region (SMR)^12^, resulting in a year-round crop.

The oomycete *Phytophthora infestans,* the causal agent of the late blight disease of potato and tomato, is the most devastating pathogen worldwide. Annual economic losses are estimated to exceed $6.7 billion in potatoes alone in the world^13^. In China, the late blight disease is the main factor constraining the sustainability of potato production^14^, particularly in the SMR where nearly 40% of China’s potatoes are grown. In recent decades, the occurrence and severity of potato late blight in China has intensified, possibly associated with the emergence of new physiological races able to overcome the main commercial cultivars currently in use, and the invasion of a novel mating type^15^ that has increased the evolutionary potential of the pathogen.

*P. infestans* is considered to be a heterothallic organism with two mating types designated as A1 and A2, despite the existence of self-fertile isolates^16^. Before the 1980s, the A2 mating type was only found in Mexico. Since then it has been detected in many parts of the world including China although tremendous variation in its frequency exists geographically^17^,^18^,^19^. Self-fertile isolates have existed at low frequency in nature for a long time^20^ but recent surveys indicate that they have now emerged as a primary mating type in some parts of the world^19^,^21^,^22^,^23^. Though many previous studies of *P. infestans* have monitored frequency changes in mating types and from this inferred the mating system and evolutionary potential of the pathogen, few studies have been conducted to determine whether other biological causes are also driving the frequency changes. Therefore, the objectives of this study were to redress this omission by: 1) studying the spatial distribution of mating types in *P. infestans* populations in China; and 2) determining the mechanisms driving changes in mating type frequency by comparing their life history traits–specifically aggressiveness, *in vitro* growth rate and tolerance to some commercial fungicides.

## Results

### Distribution and homogeneity test of mating type frequency

The mating type of *P. infestans* isolates collected from 15 provinces in China (Table 1) was determined *in vitro* by pairing individual isolates with reference testers on rye B media. These assessments showed a highly skewed mating type frequency in the *P. infestans* populations (Table 1). Among the 2250 isolates assayed, 809 (36%) produced oospores in the presence of the A2 tester strain but not on their own and were therefore scored as A1 mating type. The remaining 1441 isolates produced oospores on their own as well as in the presence of the A1 and A2 tester strains and were scored as self-fertile mating type. No A2 isolates were detected in the samples (Table 1).

Skewed and significant differences in mating type frequency were also found when *P. infestans* from different provinces or cropping regions were considered separately (Table 1). Self-fertile isolates dominated the majority of *P. infestans* populations sampled from the NSR, SMR and SWR. All isolates from Yunnan and Ningxia were self-fertile, while no self-fertile isolates were detected in Hunan or Shanxi. The average frequency of self-fertile isolates in the populations sampled from the SMR, NSR, SWR and CDR was 92%, 62%, 53% and 17%, respectively. In contrast, the A1 mating type dominated all populations sampled from the CDR. Its frequency in the four cropping regions ranged from 8% in the SMR to 83% in the CDR.

### Aggressiveness in self-fertile and A1 mating types

**Figure 1:**
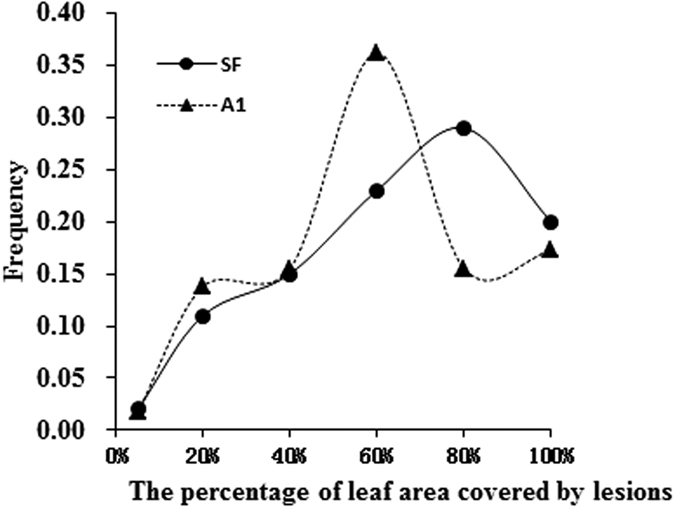
Frequency distribution of the percentage of leaf area covered by lesions (PLACL) in 82 self-fertile and 58 A1 isolates of *Phytophthora infestans* from China. Data were grouped using a binning approach and their frequency distributions were labeled with the mid-point value of the lower and upper boundaries of the corresponding bins.

The aggressiveness of a subset of the *P. infestans* isolates (140 genotypes) was determined by measuring the Percentage of Leaf Area Covered by Lesions (PLACL) on a susceptible cultivar using a detached leaflet assay. PLACL of the pathogen isolates ranged from 3.52% to 99.84% with an average of 55.1% in self-fertile isolates and from 4.3% to 97.7% with an average of 51.5% in the A1 mating type (Fig. 1). Both self-fertile and A1 mating types displayed a similar unimodal distribution in aggressiveness although the distribution in the self-fertile isolates was slightly shifted towards higher aggressiveness.

### Growth rate and tolerance to azoxystrobin and iprovalicarb in self-fertile and A1 mating types

**Figure 2:**
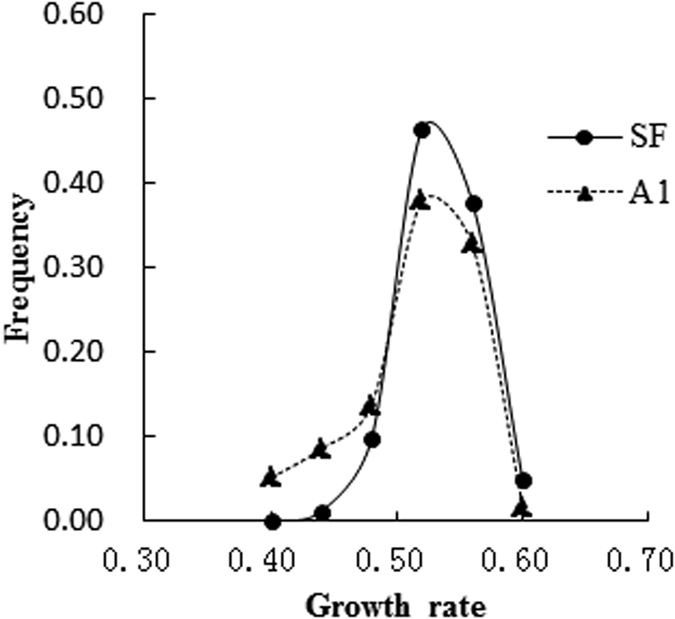
Frequency distribution of growth rate in 82 self-fertile and 58 A1 isolates of *Phytophthora infestans* from China. Data were grouped using a binning approach and their frequency distributions were labeled with the mid-point value of the lower and upper boundaries of the corresponding bins.

**Figure 3:**
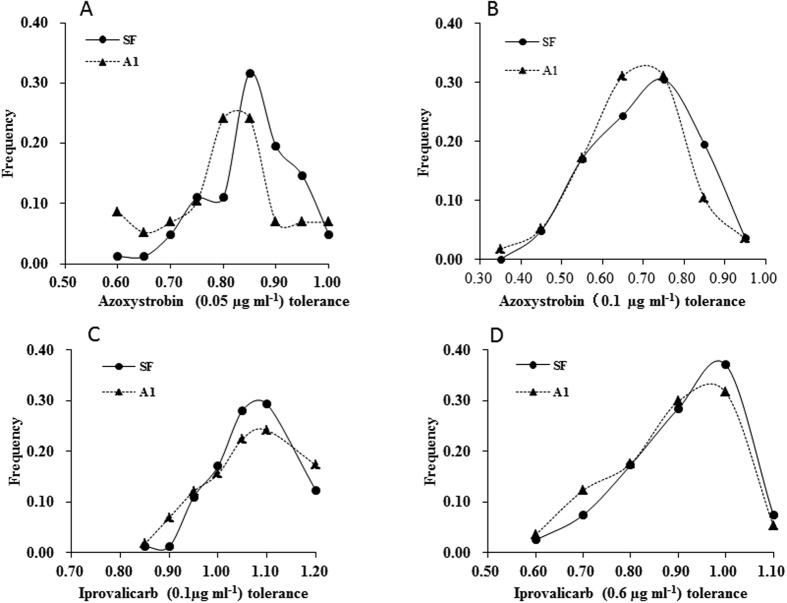
Frequency distribution of fungicide tolerance in the self-fertile and A1 isolates of *Phytophthora infestans* sampled from China. Tolerance was determined by calculating the relative growth rate of an isolate grown on rye B agar with and without supplementation of azoxystrobin and iprovalicarb: (**A**) 0.05 μg ml^−1^ azoxystrobin; (**B**) 0.10 μg ml^−1^ azoxystrobin; (**C**) 0.10 μg ml^−1^ iprovalicarb; and (**D**) 0.60 μg ml^−1^ iprovalicarb.

Growth rates of the 140 *P. infestans* genotypes were determined *in vitro* on agar in the absence of fungicides using an exponential model based on a series of measurements of colony size over time. They ranged from 0.417 to 0.572 with an average of 0.515 cm^2^/day in the self-fertile mating type and ranged from 0.376 to 0.577 with an average of 0.495 cm^2^/day in the A1 mating type (Fig. 2). Thought both mating types displayed a similar unimodal distribution and shared a common mode, A1 isolates were slightly over represented by the slower growing phenotypes (Fig. 2). Tolerance to fungicides was determined by dividing the growth rate in the presence of azoxystrobin and iprovalicarb by that in their absence. Patterns of tolerance to different concentrations of azoxystrobin were similar for the self-fertile and A1 mating types (Fig. 3), both following a unimodal distribution. At a concentration of 0.05 μg ml^−1^, tolerance to azoxystrobin among the self-fertile isolates ranged from 0.599 to 1.038 with an average of 0.835 cm^2^/day while that for the A1 mating type isolates ranged from 0.469 to 0.981 with an average of 0.781 cm^2^/day (Fig. 3A). At 0.10 μg/ml, tolerance to azoxystrobin in the self-fertile isolates ranged from 0.411 to 0.853 with an average of 0.655 cm^2^/day while that in the A1 mating type ranged from 0.315 to 0.898 with an average of 0.629 cm^2^/day (Fig. 3B).

Similar to azoxystrobin, the patterns of tolerance to different concentration of iprovalicarb also displayed a unimodal distribution in both self-fertile and A1 mating types. At a concentration of 0.1 μg ml^−1^, tolerance to iprovalicarb in the self-fertile isolates ranged from 0.846 to 1.221 with an average of 1.030 cm^2^/day and that in the A1 mating type isolates ranged from 0.844 to 1.205 with an average of 1.022 cm^2^/day (Fig. 3C). At the concentration of 0.6 μg ml^−1^, tolerance to iprovalicarb in the self-fertile isolates ranged from 0.490 to 1.081 with an average of 0.866 cm^2^/day and that in the A1 mating type ranged from 0.531 to 1.070 with an average of 0.846 cm^2^/day (Fig. 3D).

### Virulence complexity in self-fertile and A1 mating types

**Figure 4:**
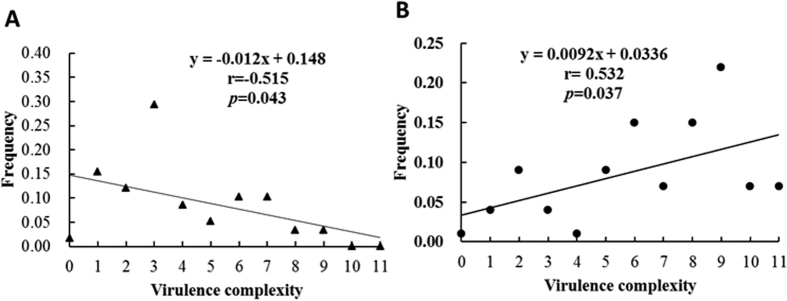
Correlation between virulence complexity and its observed frequency in the populations of *Phytophthora infestans* from China: (**A**) A1 isolates; and (**B**) self-fertile isolates.

Virulence profile for each of the 140 isolates was determined on 11 differential potato cultivars each carrying one of the 11 R genes derived from *Solanum demissum* and one universal susceptible cultivar using a detached leaf approach. The virulence complexity of an isolate was determined by the number of differential cultivars on which the isolate could induce late blight. Virulence complexity of the 140 *P. infestans* genotypes evaluated ranged from 0 to 11 with an average of 6.87 in self-fertile isolates and from 0 to 9 with an average of 3.81 in A1 mating type isolates. The virulence complexity was positively correlated with the frequency of an observed self-fertile isolate, but negatively correlated with the frequency of an observed A1 mating type isolate (Fig. 4).

### T-tests for difference in growth rate, aggressiveness, virulence complexity and fungicide tolerance between self-fertile and A1 mating types

There were significant differences in growth rate on rye B media, aggressiveness, virulence complexity and tolerance to azoxystrobin and iprovalicarb between self-fertile and A1 mating types (Table 2). On average, self-fertile isolates displayed higher growth rates, greater aggressiveness and higher virulence complexity than A1 mating type isolates (Table 3). Except at a lower iprovalicarb concentration (0.10 μg ml^−1^), self-fertile isolates on average also displayed a significantly higher fungicide tolerance than A1 isolates (Table 4).

## Discussion

Substantial research has been conducted to quantify the spatiotemporal distribution of mating types in *P. infestans* with the ultimate goals of understanding the evolutionary potential and trajectory of the pathogen^15^,^24^,^25^,^26^ and to provide information for sustainable late blight management^4^,^5^. The outcomes of these studies and the inferences that can be made are usually tempered by the small population sizes involved. For population studies, results drawn from small sample sizes are less reliable particularly for pathogens with low genotypic variation resulting from the domination of clonal lineages such as *P. infestans* in China^19^. In this study, we used a large sample size composing of many field collections to increase the statistical confidence of our results. At the same time, the hierarchical sampling method enables us to analyze the geographical distribution of mating types over different spatial scales.

Unexpectedly, although our sampling was conducted over four years, we did not detect any A2 isolates in more than 2000 isolates of *P. infestans* collected across the main potato production areas of China. This result contrasts with previous reports. Though tremendous variations exist and generally lower than the frequency of A1 counterpart^14^,^15^,^27^, the A2 mating type has been detected in many parts of China previously. Several factors may contribute to the difference between the current and previous results. It may reflect temporal and/or spatial differences in pathogen collections, a common phenomenon in the plant pathogens which are usually characterized by meta-population structure^28^. Most samples with intermediate or high frequencies of A2 mating type reported previously were collected before 2010 or nursery^23^. In contrast, all of our isolates were collected after 2010 from commercial fields. Sample sizes and analytical approaches can have an important impact on the estimate of population genetic parameters such as mating types, particularly for pathogens dominated by asexual reproduction^19^. Many of previous studies used relatively small sample sizes and data were usually not clone-corrected, possibly resulting in an overrepresentation of dominant genotypes with A2 mating type such as Blue 13-A2^29^. In the current study, sample size is large for most populations (Table 1). Though genotyping data are not available for all isolates assayed for mating types, both results from all and the 140 isolates each with different genotypes show the frequency of self-fertile mating type is ~0.60 (Tables 1 and 3). The fact that we found no A2 isolates in more than 2000 isolates suggests this mating type is now in very low frequency^27^ or may even have disappeared from most Chinese populations of *P. infestans* in recent years. It is likely that A2 mating type might have an overall lower fitness than its A1 and self-fertile counterparts (see below for the discussion of fitness differences among mating types). In a field experiment, we artificially inoculated potatoes with an equal proportion of A1 and A2 mating types each represented by three SSR-tagged genotypes. We recovered the pathogen after 8 weeks of inoculation and found that most of isolates recovered were A1 mating type (unpublished data).

Instead, a great proportion of the isolates in our collections was self-fertile with the ability to form oospores either on their own or in contact with either A1 or A2 isolates. Self-fertile isolates dominated in ~50% populations and all but one (CDR) cropping region with an overall China-wide frequency of 64%. Within individual potato growing regions the relative frequency of A1 versus self-fertile isolates in NSR and SWR showed some variation. In this respect seed potato material used in SWR typically comes from different potato breeding companies in NSR, and thus would appear to reflect the relative frequency of mating types found there.

Though we were unable to detect a directional change of frequency in the populations with multiple-year collections (data not shown), possibly due to the relatively short time interval, the finding of high frequencies of self-fertile isolates in many populations is consistent with recent reports from China^19^,^21^,^23^ and other parts of the world^22^,^30^ and is consistent with the rapid emergence of self-fertile isolates in the global population of *P. infestans*. For example, in the central highlands of Mexico, the frequency of self-fertile isolates in *P. infestans* populations collected between 2008 and 2010 was 76.1%^30^. In Gansu province of China, the frequency of self-fertile isolates in the *P. infestans* population increased from 0.00% in 2004 (none out of 21 isolates) to 17.6% in 2007 (15 out of 85 isolates)^21^. In the current study, 93% of the isolates collected from this area were classified as self-fertile (Table 1).

The skewed frequency in *P. infestans* mating types might be related to differences in their adaptation to biotic and abiotic environments coupled with nonrandom mating in the pathogen from this region^19^. Indeed, we found that on average, self-fertile isolates demonstrated higher aggressiveness (7.00%), higher fungicide tolerance (0.78–6.91%) and faster growth (4.04%) as compared to A1 isolates, suggesting a higher fitness than their counterpart. Though the differences between the two mating types in these traits are small, most of them are statistically significant (Tables 3 and 4) and could make a great contribution to the temporal population dynamics of mating frequencies. For example, assuming self-fertile mating type has 5% higher fitness than A1 mating type, its frequency would increase from 0.20 to 0.76 within 50 generations, which may only take a few years for a fast epidemic pathogen such as *P. infestans.* However, it should be aware that in addition to growth rate, fungicide tolerance and aggressiveness, the fitness of pathogens is also affected by many other ecological and life-history traits. Further study involved with more ecological and life-history traits is required to confirm the hypothesis of fitness difference between the two mating types.

Pleiotropic effects could be one factor causing this difference in fitness among mating types. Pleiotropy occurs when a single gene influences the expression of multiple phenotypic traits. Previous studies have demonstrated that, in addition to their primary role in determining the reproductive process, mating type genes are also associated with other biological functions such as maintenance of cell wall integrity^31^, aggressiveness^32^ and hyphal formation^10^. Genetic variation is low in these populations of *P. infestans*. Linkage disequilibrium could be another factor responsible for the observed differences in fitness among the two mating types. Only ~3 alleles were detected at each locus and many *P. infestans* genotypes in the current study varied by only 1–2 alleles across eight SSR loci assayed^33^, suggesting they have probably descended through mutation from just a few genetic backgrounds. Possibly, the self-fertile isolates are strongly linked with clones of a higher fitness. If this is the case then the increased frequency in self-fertile isolates may be due to hitchhiking selection^34^ for fitter genetic backgrounds.

The most interesting result in the current study is found in the association analysis showing different patterns of correlation between virulence complexity and frequency distribution in self-fertile and A1 mating types. We detected a negative correlation between the virulence complexity and frequency in the A1 mating type, consistent with the hypothesis of the existence of a fitness cost for pathogens carrying more virulence factors^26^,^35^. However, the correlation between the virulence complexity and frequency in the self-fertile isolates is positive. This result is unexpected and suggests a better performance of more complex pathogen races with a self-fertile background. Though the evolutionary mechanisms accounting for the better performance for greater virulence complexity in self-fertile isolates of *P. infestans* are not clear, the phenomenon of gradual increase in virulence complexity has also been reported in other pathogens including *Rhynchosporium secalis*^36^, *Erysiphe fischeri*^37^ and *Melampsora lini*^38^ though it is not clear whether the dynamic pattern of virulence complexity in these pathogens is mating type dependent as documented in the current study. While it has been demonstrated that many agricultural practices, such as the extensive use of R gene pyramids, cultivar mixtures, or breeding materials from composite crosses can cause selection favoring complex races^36^, these practices do not exist in potato cultivation in China. In this country, the late blight is primarily controlled by spraying fungicides or using quantitative and/or simple R genes from *S. demissum.*

Though marginally, significantly faster growth, higher aggressiveness and greater tolerance to antimicrobials in self-fertile isolates comparable to their A1 counterparts, coupled with the positive association between virulence complexity and self-fertile frequency, may have many practical implications for sustainable potato late blight control in agro-ecosystems. It suggests that genotypes with enhanced aggressiveness and resistance to antimicrobials could be generated by mutation, occasional sexual reproduction or other mechanisms and selected for under continuous and widespread use of the same resistant cultivars or antimicrobials. In this case, dynamic disease management strategies^4^,^5^ such as crop rotation, R-gene rotation, R-gene rotation combined with pyramiding should be utilized to promote diversifying selection.

## Materials and Methods

### *Phytophthora infestans* collection

Pathogen isolates were collected from 61 fields located in 15 provinces across the four major potato production zones of China between 2010 and 2013 (Table 1). For all collections, infected leaves were sampled at more than 2 m intervals from different parts of a field with at least 30 infected leaves being collected from each field. Each infected leaf was packed in a separate sandwich bag and sent to the laboratory for isolation where a single pathogen strain was isolated from each leaf. Single-spore isolates were maintained as axenic cultures on rye B agar as described previously^19^. All isolates were tested for mating types (see next section). A sub-set of isolates was genotyped with eight SSR markers as described previously^33^ and 140 isolates each with a different molecular profile^33^ were randomly selected for biological assays of growth rate, aggressiveness, virulence complexity and fungicide sensitivity as described below. The details for pathogen isolation and molecular characterizations of these populations can be found in our previous publications^19^,^33^,^39^.

### Mating type test

Mating type was determined *in vitro* as described previously^27^. Briefly, each *P. infestans* isolate was paired with an A1 tester (US970001), an A2 (US940480) or grown alone on rye B agar plates supplemented with ampicillin (100 μg ml^−1^) and rifampin (10 μg ml^−1^). After inoculation, agar plates were held at 18 °C in the dark for 12–15 days. When colonies from testers and tested isolates met, a piece of tissue was cut from the intersecting edge of the two colonies and observed microscopically to determine the production of oospores. Isolates forming oospores with the A2 tester were designated as A1 mating type. Similarly, isolates forming oospores when paired with the A1 tester were designated as A2 mating type. Isolates forming oospores with both testers and on their own were designated as self-fertile. Mating type determination was replicated three times for each isolate.

### Aggressiveness test

Aggressiveness was tested on the potato cultivar Bintje, a cultivar susceptible to all known races of *P. infestans*, using a detached leaflet assay^40^. In this assay each isolate was cultured on a rye B agar plate at 18 °C in the dark. Sporangia were harvested after 14 days by scraping the culture with a glass rod after flooding the plates with ~2 ml of chilled sterile distilled water. Sporangial suspensions were adjusted to a concentration of 5 × 10^4^ sporangia ml^−1^ using a Fuchs-Rosenthal haemocytometer and kept at 4 °C for 3–4 hours to promote zoospore release before inoculation. Fully expanded leaflets excised from Bintje plants grown in a glasshouse for 8 weeks were drop-inoculated with 5 μl of the calibrated sporangial suspensions and kept on 2% water-agar plates at 18 °C in an incubator supplemented with 16 h light daily. Diseased leaves were digitized seven days post inoculation and the area occupied by lesions was measured electronically with the image analysis software Assess^41^. Aggressiveness tests were repeated five times for each isolate.

### Virulence test

Virulence was tested by the detached leaf assayed as described previously^26^. Specifically, virulence was evaluated by inoculating spores of isolates individually onto 11 differential potato cultivars each expressing one of the R1-R11 known R genes from *Solanum demissum*^42^ and one universal susceptible cultivar (Bintje) using the same inoculum volume and concentrations and post-inoculation incubation conditions as described above for the aggressiveness test. Infection types were recorded seven days after inoculation and the test was replicated five times for each pair of isolate-differential interaction.

### Fungicide tolerance

*P. infestans* isolates from long-term storage were revived on rye B agar at 18 °C for 10 days. Mycelial plugs (10 mm in diameter) were taken from the margin of revived colonies and inoculated onto new rye B plates supplemented with either azoxystrobin (Sigma, Aldrich), iprovalicarb (Sigma, Aldrich) or without the fungicides (controls). Azoxystrobin concentrations used in the experiment were 0.05 and 0.10 μg ml^−1^ while those for iprovalicarb were 0.1 and 0.6 μg ml^−1^. Preliminary experiments indicated that these doses yielded the best result in differentiating azoxystrobin or iprovalicarb sensitivity among isolates. Many isolates did not grow when a higher dose was used while growth rates in many isolates were not significantly changed at a lower dose. The inoculated plates were kept at 18 °C in the dark and resulted colonies were photographed in 3, 5, 7, 8 and 9 days after inoculation. Colony sizes were measured using the image analysis software Assess. All treatments including controls were replicated three times. More details for the fungicide tolerance test were described previously^33^.

### Data analyses

To increase statistical power, isolates from different fields of the same province were pooled together to form a larger population. Mating type frequency was tabulated according to their geographic locations and its heterogeneity within cropping regions was evaluated with a contingency χ^2^ test^43^. Growth rates of the isolates in fungicide treatments and controls were estimated using an exponential model based on the sizes (areas) of individual colonies quantified at each point in time over the experiment. Tolerance to azoxystrobin and iprovalicarb was rated by dividing the growth rate in the presence of fungicides by that in the absence of the two fungicides as described previously^44^. Aggressiveness was measured as the percentage of leaf area covered by lesions (PLACL)^45^. Aggressiveness, growth rate and fungicide tolerance in each of the mating type categories (A1 and self-fertile) were grouped using a binning approach commonly used to display the percentile of continuous data^46^,^47^ in biological and other studies and frequency distributions were labeled graphically with the mid-point value of the lower and upper boundaries of the corresponding bins^48^. To construct the histograms, data are split into bins with each bin representing an interval of continuous data starting from the smallest bin. Each bin contains the number of occurrences in the data set that are within the corresponding bin. For example, to construct the histogram of growth rate (Fig. 1), data were split into six bins with each bin representing a 0.05 interval starting from 0.40.

Virulence profiles with respect to the 11 differentials and the universally susceptible cultivar were used to determine the race type of the isolate. An isolate was considered to carry a virulence factor towards a particular R gene (differential cultivar) if it induced a late blight lesion. The virulence complexity of an isolate was determined according to the number of differential cultivars on which the isolate induced disease^36^. A complexity index of “11” indicates that the isolate can infect all 11 differential cultivars while a complexity index of “0” indicates that the isolate is able to induce late blight disease on the universally susceptible cultivar only. All isolates carrying the same number of virulence factors were treated as having the same virulence complexity regardless of the combination of differential cultivars to which they showed virulence^36^.

Analysis of variance on growth rate, aggressiveness, virulence complexity and fungicide tolerance was performed using the general linear model procedure implemented in SAS 6.03 (SAS Institute) by treating “mating type as a fixed effect and “isolate” as a random effect and PLACL data were square root transformed. T-test^46^ was used to compare growth rate, aggressiveness, virulence complexity and fungicide tolerance between mating type groups and Pearson correlation^49^ was used to evaluate the association between virulence complexity and its observed frequency in each of the mating type groups.

## Additional Information

**How to cite this article**: Zhu, W. *et al*. Increased frequency of self-fertile isolates in *Phytophthora infestans* may attribute to their higher fitness relative to the A1 isolates. *Sci. Rep.* **6**, 29428; doi: 10.1038/srep29428 (2016).

## Figures and Tables

**Table 1:** Spatial distribution and homogeneity test of mating type frequency in the *Phytophthora infestans* populations sampled from 15 provinces across the Northern Single-cropping region (MSR), Central Double-cropping region (CDR), Southwestern Multiple-cropping region (SMR) and Southern Winter-cropping region (SWR) in China.

Region/Province	Fields	Years	Sample size	Frequency			Within region variation (χ^2^-test)
				A1	A2	Self-fertile	
NSR							
Heilongjiang	1	2013	33	0.36	0.00	0.64	341.49
Inner Mongolia	3	2013	67	0.76	0.00	0.24	(p < 0.0001)
Shanxi	5	2013	115	1.00	0.00	0.00	
Gansu	5	2010	182	0.07	0.00	0.93	
Ningxia	5	2010	100	0.00	0.00	1.00	
Subtotal			497	0.38	0.00	0.62	
CDR							
Henan	1	2012	46	0.78	0.00	0.22	31.67
Shandong	3	2012	109	0.84	0.00	0.16	(p < 0.0001)
Hubei	3	2010, 2012	117	0.79	0.00	0.21	
Hunan	1	2012	25	1.00	0.00	0.00	
Subtotal			297	0.83	0.00	0.17	
SMR							
Yunnan	5	2010, 2012, 2013	367	0.00	0.00	1.00	57.73
Guizhou	8	2011, 2012	244	0.16	0.00	0.84	(p < 0.0001)
Chongqing	11	2010, 2012	182	0.12	0.00	0.88	
Subtotal			793	0.08	0.00	0.92	
SWR							
Fujian	8	2010–2012	534	0.37	0.00	0.63	120.88
Guangdong	1	2011	73	0.97	0.00	0.03	(p < 0.0001)
Guangxi	1	2011	56	0.80	0.00	0.20	
Subtotal			663	0.47	0.00	0.53	
Total	61		2250	0.36	0.00	0.64	

**Table 2:** Analysis of variance (ANOVA) in growth rate, aggressiveness (PLACL), virulence complexity and fungicide tolerance of 140 *Phytophthora infestans* isolates sampled across China.

Trait	Source	DF	SS	MS	*F*	*P*
Growth rate	Mating type	1	0.0443	0.0443	122.46	<0.0001
	Isolate	138	0.6039	0.0043	12.09	<0.0001
	Error	280	0.1014	0.0004		
PLACL	Mating type	1	0.1431	0.1431	4.73	0.0313
	Isolate	138	8.4003	0.0689	2.01	0.0003
	Error	85	2.5742	0.0303		
Virulence complexity	Mating type	1	317.1618	317.1618	45.38	<0.0001
	Error	138	964.4381	6.9887		
Fungicide tolerance	Mating type	1	0.2983	0.2983	27.93	<0.0001
	Isolate	138	5.1760	0.0375	3.51	<0.0001
	Concentration	2	0.4018	0.2009	18.81	<0.0001
	Fungicide	1	30.5119	30.5119	2856.81	<0.0001
	Error	1525	16.2876	0.0107		

**Table 3:** Two-tailed T-test for the difference of growth rate, aggressiveness (PLACL), and virulence complexity between self-fertile and A1 isolates.

Mating type	Isolate No	Growth rate	PLACL (%)	Virulence complexity
Self-fertile	82	0.515	55.1	6.87
A1	58	0.495	51.5	3.81
Difference		4.04%	7.00%	80.31%
T-values		3.807	2.092	6.758
P-values		0.0001	0.038	<0.0001

**Table 4:** Two-tailed T-test for the difference in fungicide tolerance between self-fertile and A1 isolates.

Mating type	azoxystrobin (μg ml^−1^)		iprovalicarb (μg ml^−1^)	
	0.05	0.1	0.1	0.6
Self-fertile	0.835	0.655	1.030	0.867
A1	0.781	0.629	1.022	0.846
Difference	6.91%	4.13%	0.78%	2.48%
T-values	3.257	1.984	0.641	2.244
P-values	0.001	0.049	0.525	0.026

Data were generated from 140 distinct genotypes in which 82 were self-fertile isolates and 58 were A1 isolates.
